# Potential Novel Prediction of TMJ-OA: MiR-140-5p Regulates Inflammation Through Smad/TGF-β Signaling

**DOI:** 10.3389/fphar.2019.00015

**Published:** 2019-01-23

**Authors:** Weihao Li, Shurong Zhao, Hefeng Yang, Chao Zhang, Qiang Kang, Jie Deng, Yanhua Xu, Yu Ding, Song Li

**Affiliations:** ^1^Department of Dental Research, School of Stomatology, Kunming Medical University, Kunming, China; ^2^School of Public Health, Kunming Medical University, Kunming, China; ^3^Department of Hepatobiliary Surgery, The Second Affiliated Hospital of Kunming Medical University, Kunming, China; ^4^Department of Oral Biology and Pathology, School of Dental Medicine, Stony Brook, NY, United States

**Keywords:** mirna-140-5p, TMJ-OA, inflammation, TGF-β, Smad

## Abstract

Temporomandibular joint osteoarthritis (TMJ-OA), mainly exhibit extracellular matrix loss and condylar cartilage degradation, is the most common chronic and degenerative maxillofacial osteoarthritis; however, no efficient therapy for TMJ-OA exists due to the poor understanding of its pathological progression. MicroRNA (miR)-140-5p is a novel non-coding microRNAs (miRNAs) that expressed in osteoarthritis specifically. To investigate the molecular mechanisms of miR-140-5p in TMJ-OA, primary mandibular condylar chondrocytes (MCCs) from C57BL/6N mice were treated with interleukins (IL)-1β or transfected with miR-140-5p mimics or inhibitors, respectively. The expression of matrix metallopeptidase (MMP)-13, miR-140-5p, nuclear factor (NF)-kB, Smad3 and transforming growth factor (TGF)-β3 were examined by western blotting or quantitative reverse-transcription polymerase chain reaction (qRT-PCR). The interaction between the potential binding sequence of miR-140-5p and the 3′-untranslated region (3′UTR) of Smad3 mRNA was testified by dual-luciferase assay. Small Interfering RNA of Smad3 (Si-Smad3) was utilized to further identify the role of Smad3 mediated by miR-140-5p. The data showed MMP13, miR-140-5p and NF-kB increased significantly in response to IL-1β inflammatory response in MCCs, meanwhile, Smad3 and TGF-β3 reduced markedly. Moreover, transfection of miR-140-5p mimics significantly suppressed the expression of Smad3 and TGF-β3 in MCCs, while miR-140-5p inhibitors acted in a converse manner. As the luciferase reporter of Smad3 mRNA observed active interaction with miR-140-5p, Smad3 was identified as a direct target of miR-140-5p. Additionally, the expression of TGF-β3 was regulated upon the activation of Smad3. Together, these data suggested that miR-140-5p may play a role in regulating mandibular condylar cartilage homeostasis and potentially serve as a novel prognostic factor of TMJ-OA-like pathology.

## Introduction

Temporomandibular joint osteoarthritis is a common condition in temporomandibular joint disorder (TMD). Approximately 40% of TMD patients presented TMJ-OA (Paniagua et al., 2017). Mainly exhibit extracellular matrix loss, condylar cartilage degradation and synovial inflammation, TMJ-OA patients often accompanied with X-ray signs in clinic (Zhao et al., 2011). TMJ-OA usually accompanied by occlusal disorder which affects the patients’ life severely (Cordray, 2016). As secondary cartilage, mandibular condylar cartilage differs from other articular cartilage in certain aspects. Composed of fibrocartilage, mandibular condylar cartilage contained several thick multilayers of collagen fiber zones while other articular cartilage mainly consists of hyaline cartilage (Bouvier and Zimny, 1987). Dysregulation of chondrocyte catabolism is the key in the pathologic process of OA (Zhang et al., 2016). Previous studies reported that highly expression of type X collagen, thickening hypertrophic layer, and bone remodeling were related to the pathological alterations of TMJ-OA (Goldring, 2012; Hashimoto et al., 2013; Zhang et al., 2013; Duan et al., 2015; Zhang J. et al., 2018). Moreover, mandibular condylar cartilage seems to be more sensitive to mechanical stress (Duan et al., 2015). In spite of previous investigations, the molecular mechanism of TMJ-OA are still poorly understood. What’s more, no effective targeted therapy or early prediction for TMJ-OA exists.

Interleukins-1β is believed to be critical in cartilage damage because it can restrain matrix protein synthesis and induces proinflammatory cytokines (Kapoor et al., 2011). Previews study proved that IL-1 can cause a complex series of changes lead to synovitis, mesenchymal proliferation, and cartilage lesion. The rat OA model established by IL-1β *in vitro* showed lower levels of phosphorylated p38, phosphorylated JNK, and beta-catenin (Zeng et al., 2014). According to previous study results, IL-1β induced an efficient inflammatory response in cartilage, which resemble TMJ-OA-like change.

MicroRNA(miR/miRNAs) are novel non-coding RNAs that regulate target genes negatively. Approximately 1/3 of human mRNAs can be regulated by miRNAs, which demonstrated that miRNAs played essential roles in controlling gene expression (Lewis et al., 2005). MiRNAs inhibit its target genes by complementary binding to the 3′-untranslated regions (3′UTR) of mRNA (Liu et al., 2015). Many miRNAs have been implicated in tissue-specific or development stage-specific of various diseases, including heart disease (van Rooij et al., 2006), arthritis (Iliopoulos et al., 2008), osteoarthritis and bacterial virus infection (Liu et al., 2015). When compared with miR-140-3p, miR-140-5p seems to play more important function in the BMG rat model (Min et al., 2015). Miyaki et al. (2009) reported that miR-140 expressed largely difference between human articular chondrocytes and mesenchymal stem cells, and the expression of miR-140 found reduced in human osteoarthritis cartilage. However, Swingler et al. (2012) reported that the expression of miR-140-5p and miR-455-3p was increased in OA cartilage when compared with the cartilage of femoral neck fractures patients. These findings demonstrated that abnormal gene expression of miR-140-5p may contribute to different stages of development in OA, however, the exact regulatory mechanism of miR-140-5p is still unclear, especially in TMJ-OA.

Recent studies showed that the TGF-β superfamily play essential roles in almost all aspect of development, from the generation of germ cells to organ formation, and into postnatal life (Wang et al., 2014). TGF-β3 expressed highly in ribs and vertebral cartilage. Previews studies showed that TGF-β3 serve an important role in cartilage maintenance and homeostasis (Sun et al., 2018). The TGF-β family transfer signal transmission primarily be conducted via Smad proteins, which acting a role of receptor activation in signal transduction. Consequently, TGF-β/Smads regulate cartilage development, including chondrocytes proliferation, early differentiation, hypertrophy, terminal differentiation, and maintenance of homeostasis (Sun et al., 2016). Interestingly, no research has completely clarified the regulatory mechanism of microRNA in TMJ-OA. In the present study, we identified that Smad3 is the direct target of miR-140-5p. Dysregulation of miR-140-5p contributes to TMJ-OA pathogenesis through Smad/TGF-β signaling.

## Materials and Methods

### MCCs Preparation

C57BL/6N mice (2-week-old, male, 5 g) were obtained from Model Animal Research Center of Kunming Medical University (Yunnan, China). This study was carried out in accordance with the recommendations of Chinese Academy of Sciences. Mice mandibular condylar cartilage were cut into tiny fragments, then digested with 0.25% trypsin (Gibco Invirtrogen, United States) for 25 min at 37°C and 0.2% collagenase (Sigma-Aldrich, United States) for 45min at 37°C. Followed by dissociation and filtration, cells were centrifuged at 1200 rpm for 5min. Primary MCCs were cultured according to previous method (Gosset et al., 2008). Freshly isolated at passage 1 (P1), MCCs were placed in monolayer culture in 6-well plates (for RNA and protein) with DMEM/F-12 (Gibco Invirtrogen, United States) medium containing 10% fetal bovine serum (Gibco Invirtrogen, United States). After serum starved for 12 h, MCCs were induced by 10 ng/ml IL-1β (R&D systems, Minneapolis, United States) stimulation for 24 h, then treated with or without transfection experiments, respectively. Every 3 days, medium in the 6-well plates was refreshed for once.

### MCCs Transfection

miR-140-5p mimics, miR-140-5p inhibitors, and their corresponding negative controls were designed and obtained from RiboBio (RiboBio, Guangzhou, China). Cultured and plated in 6-well plates (∼50% confluence), MCCs were starved for 12 h, followed by 48 h transfection of miR-140-5p mimics or inhibitors, then treated with IL-1β for 24 h, respectively. MCCs transfected with microRNA mimics or inhibitors of miR-140-5p at a concentration of 50 nM by using riboFECTTM CP Transfection Kit (166T) (RiboBio, Guangzhou, China) according to the manufacturer’s protocol.

### Immunofluorescence Analysis

Mandibular condylar chondrocytes were cultured on 18-mm glass coverslips (Thermo Fisher Scientific, United States) and stained for single immunofluorescence staining. Fixed in 50% (*v*/*v*) methanol/50% (*v*/*v*) acetone for 5 min. Subsequently, cells were rinsed in 1 × DPBS (Gibco Life Technologies, United States) for 3 times, each time 5 min, followed by incubation with rabbit anti-Smad3 (1:200 dilution; Abcam, Cambridge, MA, United States) at 4°C overnight. FITC-labeled goat anti-rabbit IgG (Beyotime, Shanghai, China) were used as secondary antibodies and incubate for 60 min. Cell nucleus were counterstained with DAPI (Solarbio, Beijing, China) for 10 min. Coverslips were mounted in fluorescent mounting medium (Solarbio, Beijing, China). Finally, the fluorescence micrograph were captured and analyzed by confocal laser scanning microscope system (Nikon, Co., Ltd., Tokyo, Japan).

### Dual-Luciferase Assay

miRTarBase^1^ was utilized to predict the potential binding sequence between miR-140-5p and Smad3. The wild-type (WT) or mutant-type (MUT) 3′URT of Smad3, which containing the predicted binding sequences or mutant binding sites, was cloned into pmiR-RB-REPORT^TM^ luciferase vector (RiboBio, Guangzhou, China) by XhoI/NotI restriction sites, respectively. The coding sequences of 3′UTR of Smad3 for PCR-amplified primers were as follows: 5′-TATGTTGGCTGGAAAACCACAA-3′. After amplification, the PCR products were cloned into the vector for dual-luciferase assay. At a concentration of 1.5 × 10^4,^ cells (293T) in a 96-well plate were transfected with miR-140-5p or negative control by Lipofectamine^TM^ 3000 (Invirtrogen, Carlsbad, CA, United States) and incubated for 48 h. Finally, luciferase activity was measured posttransfection using Dual-Glo^®^ Luciferase Assay System (Promega, Madison, WI, United States). The activity ratio of each group was normalized to the corresponding *Renilla* luciferase activity.

### SiRNA Smad3 Transfection

Mandibular condylar chondrocytes were transfected with small interference RNA of mouse Smad3 (Si-Smad3) to suppress the Smad3 expression. According to the protocol, the transfection were performed by riboFECTTM CP Transfection Kit (166T) (RiboBio, Guangzhou, China). After starved for 12 h, MCCs were followed by 48 h transfection of Si-Smad3.

### Quantitative Reverse-Transcription Polymerase Chain Reaction (qRT-PCR) Analysis

Following to the manufacturer’s instructions, 1 μg for total RNA were extracted by Eastep^®^ Super Total RNA Extraction Kit (Promega, Madison, WI, United States) then reversed transcribed into cDNA by A5000 GoScript^TM^ Reverse Transcription System (Promega, Madison, WI, United States). Then miRNAs were reverse transcribed into cDNA with stem-loop primers by using miDETECT A Track^TM^ miRNA qRT-PCR Starter Kit (RiboBio, Guangzhou, China). According to the manufacturer’s instructions, qRT-PCR was performed in a CFX96 system (Bio-Rad, Hercules, CA, United States) using SybrGreen qPCR Mastermix (DBI Bioscience, Ludwigshafen, Germany). β-actin and small nuclear RNA U6 were used as internal controls to normalized gene expressions. The thermal cycling condition was 95°C for 10 min, followed by 40 cycles of 95°C for 30 s and 60°C for 30 s. The relative expression of miR-140-5p to U6, and relative expression of mRNA to β-actin were measured by using the comparative 2^-ΔΔct^ method (Livak and Schmittgen, 2001). Specific primers of mouse miR-140-5p and U6 were designed by RiboBio (RiboBio, Guangzhou, China). Primer sequences used for qRT-PCR were as follows: mouse β-actin [GenBank: NM_007393.5], forward 5′-GTGCTATGTTGCTCTAGACTTCG-3, reverse 5′-ATGCCACAGGATTCCATACC-3′; mouse MMP13 [GenBank: NM_007393.5]: sense 5′-CAGTTGACAGGCTCCGAGAA-3′ and antisense 5′-CCACATCAGGCACTCCACAT-3′.

### Western Blot Analysis

Western blotting was performed as previously described (Zhang C. et al., 2018). MCCs were harvested by whole-cell lysates with ice-cold lysis buffer (1% NP-40, 50 mM Tris–HCL, 0.1%SDS, and pH 7.4 150 mM NaCl) and protease inhibitors (Beyotime, Shanghai, China). Normalized by BCA assay kit (Beyotime, Shanghai, China), proteins were separated by SDS-PAGE at 80 V for 30 min, then 120 V for 1 h. After proteins transferred, the PVDF membrane (Millipore, Billerica, MA, United States) was then blocked in 5% non-fat milk at 26°C for 1 h. Next, the blocked membrane was hybridized with primary antibodies anti-Smad3 (1:5,000 dilution; Abcam, Cambridge, MA, United States), anti-TGF-β3 (1:1,000 dilution; Abcam, Cambridge, MA, United States), anti-NF-kB (1:1,000 dilution; Abcam, Cambridge, MA, United States), anti-NF-kB2 (1:1000 dilution; Abcam, Cambridge, MA, United States) and anti-β-actin (1:5,000 dilution; Abcam, Cambridge, MA, United States) at 4°C over night, followed by secondary antibodies goat anti-rabbit IgG (1:5,000 dilution; Abcam, Cambridge, MA, United States) at 26°C for 1 h. Finally, the membrane was incubated with ECL Western blotting Kit (Beyotime, Shanghai, China). The immuno-reactivity percentages to control group was analyzed by Image J or Image Lab software provided by the Institute of Stomatology.

### Statistical Analysis

All data in the present study were represented as mean ± standard deviation (SD). Each group was repeated three times (*n* = 3). GraphPad Prism 5.0 or SPSS 17.0 were performed to statistical analyses. Student’s *t*-test was conducted when two groups compared. One-way ANOVA followed by Bonferroni *post hoc* tests was used for multiple groups comparisons. The statistically significant difference considered when *P* values < 0.05.

## Results

### The Expression of MMP13, MiR-140-5p, NF-κB Increased but Smad3, TGF-β3 Downregulated in Response to IL-1β

**FIGURE 1:**
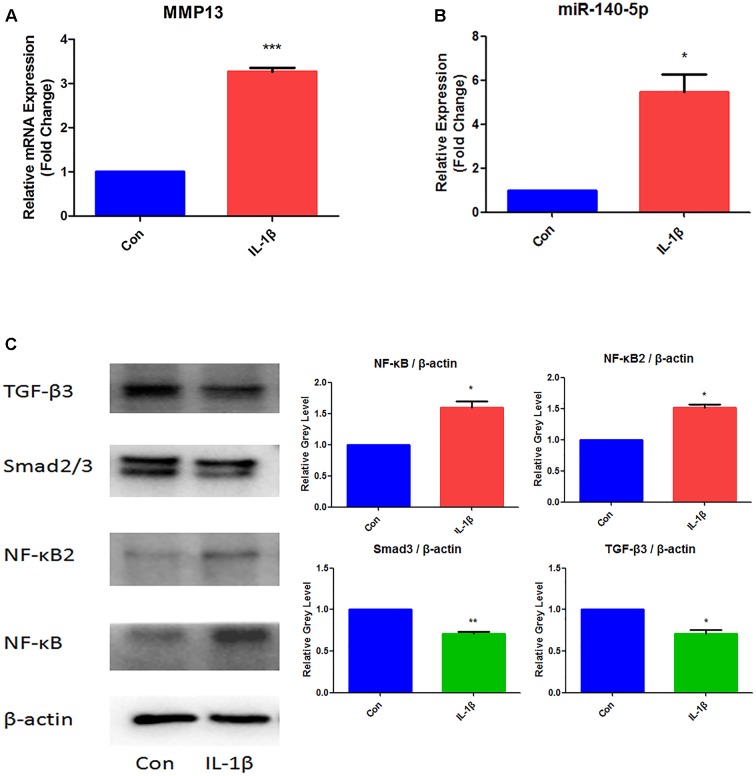
Expression analysis of MMP13, miR-140-5p, NF-κB, NF-κB2, Smad3, and TGF-β3 in IL-1β-stimulated MCCs. **(A)** Assessment of MMP13 mRNA relative expression by qRT-PCR, **(B)** Assessment of miR-140-5p relative gene expression by qRT-PCR, and **(C)** Evaluation of NF-κB, NF-κB2, Smad3, TGF-β3 protein expression. β-actin and small nuclear RNA U6 were used as controls. Each group repeated three times (*n* = 3). Control group (Con) were without IL-1β stimulation. ^∗^*P* < 0.05 when compared with Con. ^∗∗^*P* < 0.01 when compared with Con. ^∗∗∗^*P* < 0.001 when compared with Con.

In order to established TMJ-OA model *in vitro*, MCCs were induced by IL-1β (10 ng/ml) for 24 h. The effects of IL-1β were examined in Figure 1. Determined by qRT-PCR, The relative expression of MMP13 mRNA increased significantly (Figure 1A). MiR-140-5p increased significantly when compared with control group (Figure 1B). Western blot analysis results showed that IL-1β treatment significantly increased NF-κB and NF-κB2 protein level. However, treats with IL-1β effectively suppressed the expression of Smad3 and TGF-β3 (Figure 1C).

### Smad3 and TGF-β3 Downregulated via MiR-140-5p

**FIGURE 2:**
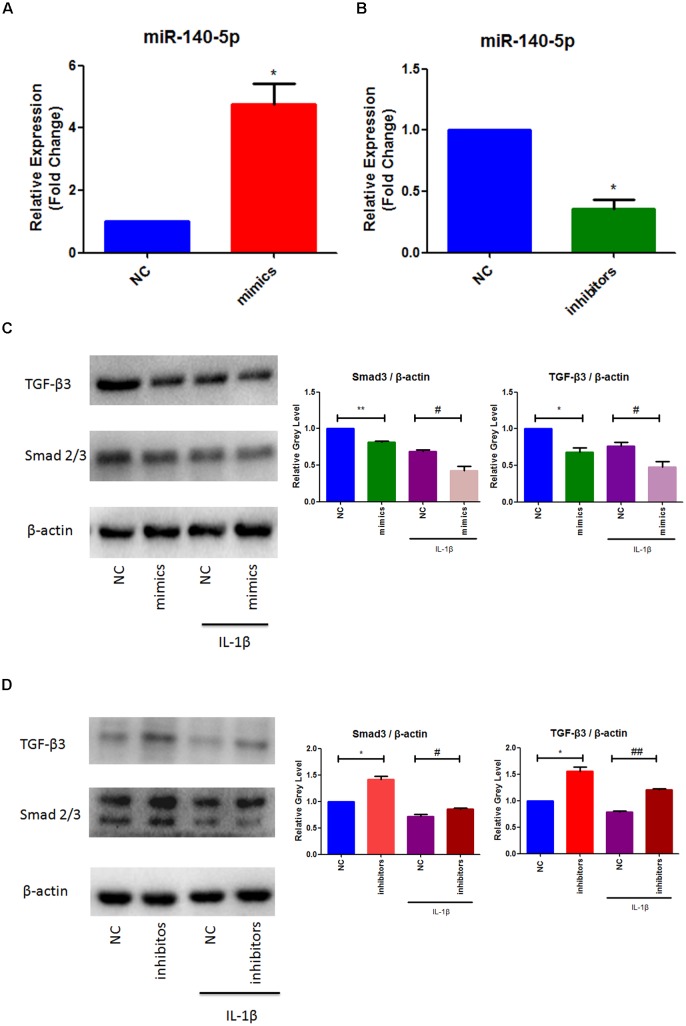
MiR-140-5p mediated Smad3 and TGF-β3 in response to IL-1β. **(A)** Evaluation relative microRNAs expression of miR-140-5p in MCCs transfected with mimics, **(B)** Evaluation relative microRNAs expression of miR-140-5p in MCCs transfected with inhibitors, **(C)** Evaluation relative protein production of Smad3 and TGF-β3 after mimics transfection and stimulation of IL-1β in MCCs, and **(D)** Evaluation relative protein production of Smad3 and TGF-β3 after inhibitors transfection and stimulation of IL-1β in MCCs. β-actin and small nuclear RNA U6 were used as controls. Each group repeated three times (*n* = 3). ^∗^*P* < 0.05 when compared with the negative control group (NC). ^∗∗^*P* < 0.01 when compared with NC. ^#^*P* < 0.05 when compared with NC induced by IL-1β. ^##^*P* < 0.01 when compared with NC induced by IL-1β.

**FIGURE 3:**
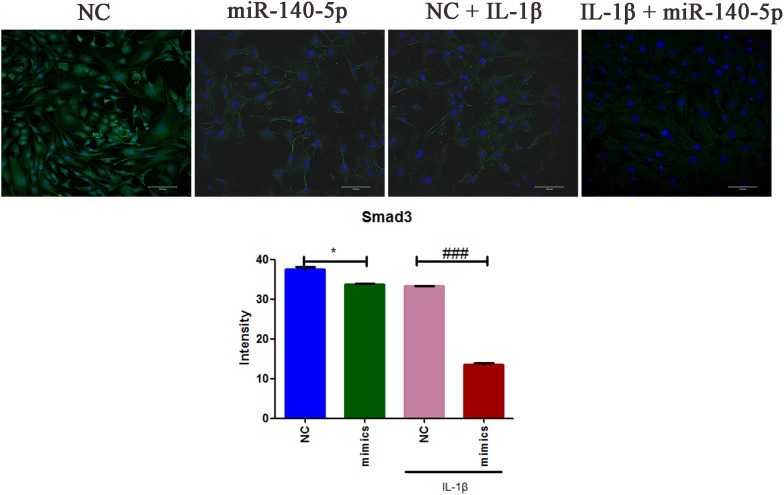
The immunofluorescence intensities analysis of Smad3 expression in MCCs. MCCs were transfected with or without miR-140-5p mimic for 48 h, then stimulated with or without IL-1β for 24 h, respectively. Scale bar = 10 μm (magnification, ×200). Typical confocal micrographs of Smad3 showed in green, and DAPI in blue. Data are represented as means ± standard deviation (*n* = 3). ^∗^*P* < 0.05 when compared with the negative control group (NC). ^###^*P* < 0.001 when compared with NC induced by IL-1β.

To further elucidate the role of miR-140-5p in TMJ-OA like damage, specific miRNA mimics and inhibitors of mouse miR-140-5p were used for transfection experiment. MCCs were transfected with miR-140-5p mimics, or treated with miR-140-5p inhibitors for 48 h, respectively, followed with or without stimulation of IL-1β for 24 h. As showed in Figure 2, miR-140-5p relative expression level was assessed by qRT-PCR. Transfection of miR-140-5p mimics increased miR-140-5p expression significantly (Figure 2A), while inhibitors acted in a converse manner (Figure 2B). Compared with negative control, overexpression of endogenous miR-140-5p significantly reduced Smad3 and TGF-β3 protein levels (Figure 2C). Conversely, miR-140-5p inhibitors increased Smad3 and TGF-β3 protein production, and counteract the stimulation of IL-1β markedly (Figure 2D). Consistent with this, the results of immunofluorescence analysis showed the similar fluorescence intensity (Green) changes of Smad3 (Figure 3). When compared with the negative control, overexpression of miR-140-5p reduced the fluorescence intensity of Smad3, especially in the presence of IL-1β-induced MCCs. Thus, miR-140-5p contributes to the alteration of Smad3 and TGF-β3 expression at protein level.

### MiR-140-5p Directly Inhibits the Expression of Smad3

**FIGURE 4:**
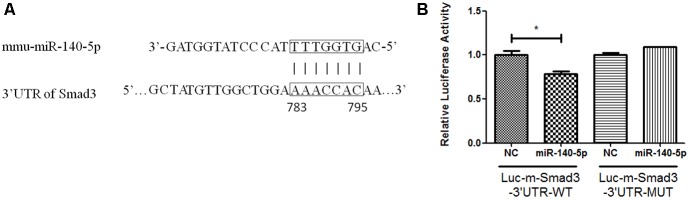
Smad3 mediated by miR-140-5p directly. **(A)** The predicted binding sites of miR-140-5p to the 3′UTR of Smad3, **(B)** Analysis of relative luciferase activity. Data were represented as means ± standard deviation (*n* = 3). ^∗^*P* < 0.05 vs. negative control group (NC) with Luc-m-Smad3-3′UTR-WT.

To investigate whether miR-140-5p regulates the expression of Smad3 directly, we identified a potential miR-140-5p binding sequence in the 3′-untranslated region (3′UTR) of Smad3 (Figure 4A) by miRNA target prediction software and checked in miRTarBase (Lewis et al., 2005). To further identify the predicted sequence in the 3′UTRs of Smad3 mRNA interacting with miR-140-5p, cells (293T) were performed transfection with luciferase reporters inserted the target fragment (Luc-m-Smad3-3′UTR-WT), or with vectors carrying mutated constructs (Luc-m-Smad3-3′UTR-MUT), or miR-140-5p or corresponding negative control (NC), respectively. Cells were harvested after 48 h transfection. Compared with NC, cells co-transfected with Luc-m-Smad3-3′UTR-WT and miR-140-5p showed lower luciferase activity. In contrast, luciferase activity examined no significant change when compared with co-transfection with miR-140-5p and Luc-m-Smad3-3′UTR-MUT, in which the predicted binding sequence to miR-140-5p were mutated (Figure 4B). Together, the data suggested that the miR-140-5p can regulated Smad3 directly.

### MiR-140-5p Induced-Downregulation of TGF-β3 Is Mediated by Smad3

**FIGURE 5:**
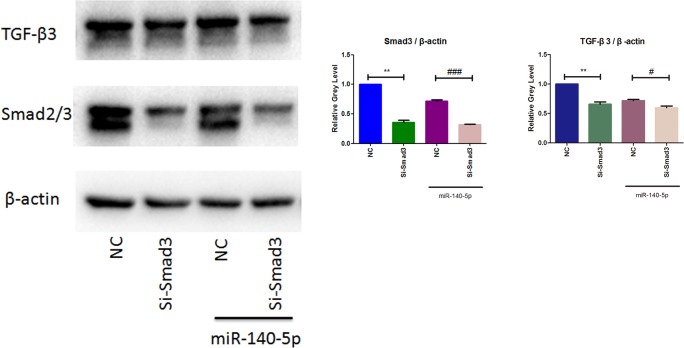
TGF-β3 regulated through miR-140-5p/Smad3 signaling. Western blotting analysis of the relative protein expression of Smad3 and TGF-β3. Data were represented as means ± standard deviation (*n* = 3). ^∗∗^*P* < 0.01 vs. negative control group (NC). ^#^*P* < 0.05 when compared with NC co-transfected with miR-140-5p. ^###^*P* < 0.001 when compared with NC co-transfected with miR-140-5p.

To identify the signaling pathway of Smad3 in mediating the downregulation of TGF-β3 was induced by miR-140-5p, we utilized small interfering RNA of Smad3 (Si-Smad3) for further transfection experiment. MCCs were co-transfected with or without Si-Smad3 in the presence or absence of miR-140-5p. Cells were harvested after 48 h transfection. As western blotting results showed in Figure 5, cells transfected with Si-Smad3 exhibit an efficiently reduction of Smad3 and TGF-β3. When compared with the miR-140-5p transfection group, cells co-transfected with Si-Smad3 and miR-140-5p blocked Smad3 expression more significantly. What’s more, miR-140-5p and Si-Smad3 showed similar effects on downregulation of TGF-β3. Interestingly, the group co-transfected with miR-140-5p and Si-Smad3 observed lower TGF-β3 expression compared to miR-140-5p group, which suggesting that miR-140-5p induced-downregulation of TGF-β3 might be more than Smad3 pathway. Together, these results indicate miR-140-5p mediates down-regulation of TGF-β3 by Smad3.

## Discussion

As widely accepted, IL-1β is a key mediator in accelerating the activity of MMPs (Ge et al., 2009). MMP13, a representative of MMPs in OA or OA-like changes (de Rooy et al., 2014; Agere et al., 2017; Li et al., 2017; Perotto et al., 2018), reported to be a factor contributes to the early stage development of OA. TMJ-OA is a common disorder in mandibular joint, however, the molecular networks have not been characterized in detail. Due to a limited prediction or therapy for TMJ-OA, the pathogenesis and regulatory mechanism of TMJ-OA urgently need further investigation. NF-κB is an efficient nuclear factor that accelerate inflammatory reaction (Jiang et al., 2018). The present data showed upregulation of MMP13 and NF-κB in MCCs, which indicated a reliable establishment of TMJ-OA model *in vitro*.

Previous study reported that TGF-β is an essential initiator of chondrogenesis. Inhibition of TGF-β caused a reduction of extracellular matrix and resulted in OA (van der Kraan et al., 2012). Smad3 proved to be a key in classic TGF-β signal conduction (Yang et al., 2001). Mutant mice of Smad3 developed joint disease like OA (van de Laar et al., 2011). Vertebral and ribs cartilage found TGF-β3 highly expressed, particularly in the proliferative and hypertrophic zones (Wang et al., 2014). In the perichondrium, TGF-β3 was also expressed at a higher level than other TGF-βs (Pelton et al., 1990). TGF-β3 enhanced the chondrocyte phenotype and differentiation (Moioli and Mao, 2006; Dahlin et al., 2014). As a result, alteration of TGF-β3 may be suitable for the evaluation of OA (Ude et al., 2018). Dysregulation of both miR-140-3p and miR-140-5p were correlated with osteoarthritis (Yin et al., 2017). Recent studies revealed some pathological characteristics of OA, but the exact way of TMJ-OA pathogenesis still need further investigation.

The present study exhibit an increased expression of miR-140-5p was found in MCCs induced by IL-1β. MiR-140-5p, originated from miR-140, was reported to be expressed in cartilage particularly. Miyaki et al. (2010) created miR-140-null mice and reported that miR-140 regulates cartilage development and homeostasis. MiR-140 loss was responsible for age-related OA. Wang et al. (2018) found that miR-140-5p/miR-149 effect on chondrocyte metabolism via FUT1. Contradictorily, Swingler et al. (2012) compared human OA cartilage with control cartilage obtained from patients with femoral neck fractures, and found that the expression miR-140-5p was increased in OA cartilage. These contradictory findings may explained by dual-function of miR-140-5p at different development stage of TMJ-OA. Alteration miR-140-5p possibly acted a role like MMP13 in chondrogenesis. Abnormal changes of miR-140-5p may suggest a process of TMJ-OA at early stage. Combined with previous literature, our observation showed a close relationship between miR-140-5p and TGF-β pathway. In the present work, we provided evidence that miR-140-5p is a trait of IL-1β-induced responsiveness in MCCs, which lead to a reduction of Smad3 and TGF-β3 at protein level. Specific miRNA mimics and inhibitors of mmu-miR-140-5p were used for transfection experiment. Overexpression of endogenous miR-140-5p significantly reduced Smad3 and TGF-β3 protein levels, but miR-140-5p inhibitors acted in a converse manner. Moreover, miR-140-5p inhibitors counteracted the IL-1β-induced suppression of Smad3 and TGF-β3 markedly. Immunofluorescence analysis ensured the conclusion again. Thus, miR-140-5p contributes to the alteration of Smad3 and TGF-β3 expression at protein level. The results indicated IL-1β affects Smad3 and TGF-β3 through miR-140-5p.

As a key transcription factor in mediating TGF-β signaling pathway, Smad3 was observed an inversely relationship to miR-140-5-p. Next, we used dual-luciferase constructs and reporter assay to identify whether miR-140-5p inhibits Smad3 expression directly. Dual-luciferase assay demonstrated that miR-140-5p interacted with predicted binding sequence in the 3′UTR of Smad3, which consistent with Pais et al.’s (2010) conclusion. Furthermore, Vincenti and Brinckerhoff (2001) reported that TGF-β may also suppressed by blocking the expression of Smad4, which found to be a competitive mediator in Smad pathway to control chondrocytes terminal differentiation (van der Kraan et al., 2012).

At last, we provided evidence to demonstrate miR-140-5p regulated the expression of TGF-β3 through Smad3. To further investigation, we utilized small interfering RNA of mouse Smad3 (Si-Smad3) to co-transfected with miR-140-5p in MCCs. Compared with negative control, respectively, both of Si-Smad3 and miR-140-5p mimics exhibit an efficiently reduction of TGF-β3. Interestingly, co-transfection of miR-140-5p mimics and Si-Smad3 exhibit a more efficient reduction of TGF-β3 protein expression. It suggested that TGF-β3 may regulated by miR-140-5p in multiple ways, including Smad3 pathway. Novel evidences demonstrating that miR-140-3p negatively controlled TGF-β3, which proved to be a direct target of miR-140-3p (Fushimi et al., 2018). That’s may explained why co-transfection of miR-140-5p mimics and Si-Smad3 showed a lower expression of TGF-β3. At least partially, these results demonstrated miR-140-5p-induced downregulation of TGF-β3 was mainly mediated by Smad3.

## Conclusion

Taken together, the present study provided evidence that miR-140-5p regulated TMJ-OA pathogenesis through TGF-β/Smad signaling pathway and might be a potential predictive factor for TMJ-OA.

## Ethics Statement

According to the recommendations of Chinese Academy of Sciences, the protocol of this study was approved by the Animal Experimental Ethics Committee of Kunming Medical University.

## Author Contributions

WL and SL designed and developed the research. SZ, HY, CZ, QK, and JD conducted the experiments, and collected and analyzed the data. YX and YD wrote and drafted the manuscript.

## Conflict of Interest Statement

The authors declare that the research was conducted in the absence of any commercial or financial relationships that could be construed as a potential conflict of interest.

## Footnotes

1 http://mirtarbase.mbc.nctu.edu.tw/php/index.php

## Abbreviations

ANOVA: analysis of variance
MCCs: mandibular condylar chondrocytes
MMP13: matrix metallopeptidase 13
NF-kB: Nuclear factor kB
PBS: phosphate-buffered saline
PVDF: polyvinylidene difluoride
qRT-PCR: quantitative reverse-transcription polymerase chain reaction
SDS-PAGE: sodium dodecyl sulfate polyacrylamide gel electrophoresis
Smad2/3: Sma-and Mad-related protein 2/3
TGF-β3: transforming growth factor-β 3
TMJ-OA: temporomandibular joint osteoarthritis
